# Targeting erythrocyte carbonic anhydrase and ^18^O-isotope of breath CO_2_ for sorting out type 1 and type 2 diabetes

**DOI:** 10.1038/srep35836

**Published:** 2016-10-21

**Authors:** Chiranjit Ghosh, Santanu Mandal, Gourab D. Banik, Abhijit Maity, Prabuddha Mukhopadhyay, Shibendu Ghosh, Manik Pradhan

**Affiliations:** 1 Department of Chemical, Biological & Macro-Molecular Sciences, S. N. Bose National Centre for Basic Sciences, Salt Lake, JD Block, Sector III, Kolkata, 700098, India; 2 Department of Medicine, Vivekananda Institute of Medical Sciences, 99 Sarat Bose Road, Kolkata, 700027, India; 3 Department of Medicine, Raipur Institute of Medical Sciences, Raipur, 492006, Chhattisgarh, India; 4 Thematic Unit of Excellence on Nanodevice Technology, S. N. Bose National Centre for Basic Sciences, Salt Lake, JD Block, Sector III, Kolkata, 700098, India; 5 Technical Research Centre, S. N. Bose National Centre for Basic Sciences, Salt Lake, JD Block, Sector III, Kolkata, 700098, India

## Abstract

The inability to envisage the acute onset and progression of type 1 diabetes (T1D) has been a major clinical stumbling block and an important area of biomedical research over the last few decades. Therefore there is a pressing need to develop a new and an effective strategy for early detection of T1D and to precisely distinguish T1D from type 2 diabetes (T2D). Here we describe the precise role of the enzymatic activity of carbonic anhydrase (CA) in erythrocytes in the pathogenesis of T1D and T2D. We show that CA activities are markedly altered during metabolism of T1D and T2D and this facilitates to the oxygen-18 (^18^O) isotopic fractionations of breath CO_2_. In our observations, T1D exhibited considerable depletions of ^18^O-isotopes of CO_2,_ whereas T2D manifested isotopic enrichments of ^18^O in breath CO_2_, thus unveiling a missing link of breath^18^O-isotopic fractionations in T1D and T2D. Our findings suggest that the alterations in erythrocytes CA activities may be the initial step of altered metabolism of T1D and T2D, and breath ^18^O-isotope regulated by the CA activity is a potential diagnostic biomarker that can selectively and precisely distinguish T1D from T2D and thus may open a potential unifying strategy for treating these diseases.

Type 1 diabetes (T1D), a chronic autoimmune disorder resulting from destruction of insulin-producing β-cells in the pancreatic islets of Langerhans, is an important and serious health problem afflicting millions of people worldwide^1^. Over the last few decades much effort has been devoted towards identifying the T1D from several measureable markers of the autoimmune state as well as the progression of islet destruction. The most commonly used indicators are glutamic acid decarboxylase autoantibodies (GADA), islet cell cytoplasmic autoantibodies (ICA), insulin autoantibodies (IAA) and insulinoma associated-2 autoantibodies (IA-2A)^2^. Although the occurrence of T1D is suggested by the presence of one or different types of antibodies, but it is still the subject of considerable debate within the healthcare community when and which antibody should be tested for precise identification of disease state. Moreover, the potential role of autoantibodies in the pathogenesis of disease is highly controversial^3^,^4^. The underlying mechanisms are also poorly understood. Sometimes, maturity onset-diabetes of young (MODY) are misdiagnosed as T1D and incorrectly treated with insulin^5^,^6^. Moreover, the autoantibody-based diagnosis of T1D is a major challenge as there remains a subset of patients who does not express the features reflective of autoantibody characteristics at the onset of disease. Therefore, the inability to visualize the acute onset and progression of T1D has been a major area of research and clinical stumbling block over the last decade. The uncertainty of autoantibodies testings suggests that there is a pressing need to develop a new and effective strategy for early detection of T1D. Indeed, to our knowledge, so far no study has reported any specific diagnostic biomarker for T1D that can accurately track the initiation and progression of the disease as well as can also be used for routine clinical practices.

Type 2 diabetes (T2D) is another form of diabetes mellitus, where body either does not produce enough insulin or body is resistant to insulin action. The occurrence of T2D is very common and it accounts for 80% of total diabetes cases. The insulin resistant individuals with genetically higher rate of β-cell apoptosis are highly susceptible for developing type 1 diabetes^7^ and consequently features of T1D and T2D may be present in the same patient, making the precise identification and classification of T1D and T2D extremely difficult. Although diabetic ketoacidosis (DKA) is a common incident for the onset of T1D, but the history of DKA is not a reliable way to distinguish between T1D and T2D. Now-a-days, C-peptide, an amino-acid polypeptide, has also been reported to be a potential candidate to distinguish the T1D from T2D^8^. Furthermore, the measurement of adiponectin-to-leptin ratio has been suggested to exhibit considerable advantages to detect T1D than the other proposed methods^9^. But, neither C-peptide nor adiponectin-to-leptin ratio has yet showed potential routine clinical applications for precise diagnosis of T1D and T2D. To our knowledge, as there is no clinical characteristic or diagnostic marker available till now to readily distinguish T1D from T2D, therefore there is a major challenge worldwide to develop a new and suitable diagnostic marker that can selectively and precisely track the progression of both T1D and T2D.

However, studies in the past decade have also demonstrated that oxygen isotopes (i.e. ^16^O and ^18^O) are rapidly interchanged between carbon dioxide (^12^C^16^O^16^O) and water (H_2_^18^O) to produce stable ^12^C^18^O^16^O isotope in exhaled breath during the human respiration process mediated by the carbonic anhydrase (CA), a widespread metalloenzyme in human body^10^,^11^. Moreover, some early evidences^11^,^12^ suggest that changes in CA activity in human erythrocytes (red blood cells) may be the initial step of altered metabolism in diabetes mellitus. But the precise role of CA activity in the pathogenesis of T1D or T2D is completely unknown. Moreover, the potential role^13^,^14^ of ^18^O-isotopic exchange regulated by CA activity suggests a tantalizing but unproven hypothesis about the possibility of exploiting breath ^18^O-isotopic fractionation may specifically track the progression of T1D and T2D, and hence may introduce a novel noninvasive strategy for an accurate and early detection of diabetes as well as to distinguish T1D specifically from T2D. A new insight into the role of CA activity linking to ^18^O-isotopic fractionations of breath CO_2_ is important to delay or even prevent the acute onset of T1D or T2D and its complications. In addition, unravelling the precise metabolic pathways underlying breath ^18^O isotopic alternation remains a challenge, whenever an individual is at high-risk of developing diabetes.

Herein, we report how the enzymatic activity of CA in human erythrocytes is altered in individuals with T1D and T2D, and subsequently we investigated how the ^18^O-isotopic fractionations of breath CO_2_ are linked to the alteration of CA activity in both T1D and T2D subjects. We also provide evidences that the breath ^12^C^18^O^16^O isotope can be utilized as a potential noninvasive biomarker for selectively distinguishing T1D cases from those with T2D. We furthermore explored several optimal diagnostic parameters of CA activity in human erythrocytes and ^18^O-isotopes in exhaled breath CO_2_ keeping an eye on the widespread clinical efficacy of our new methodology for the diagnosis and precise classification of T1D and T2D. We finally elucidated the potential metabolic pathways underlying the mechanisms linking breath ^18^O-isotopes to T1D and T2D to gain a better insight into the pathogenesis of diabetes mellitus.

## Results and Discussion

**Figure 1:**
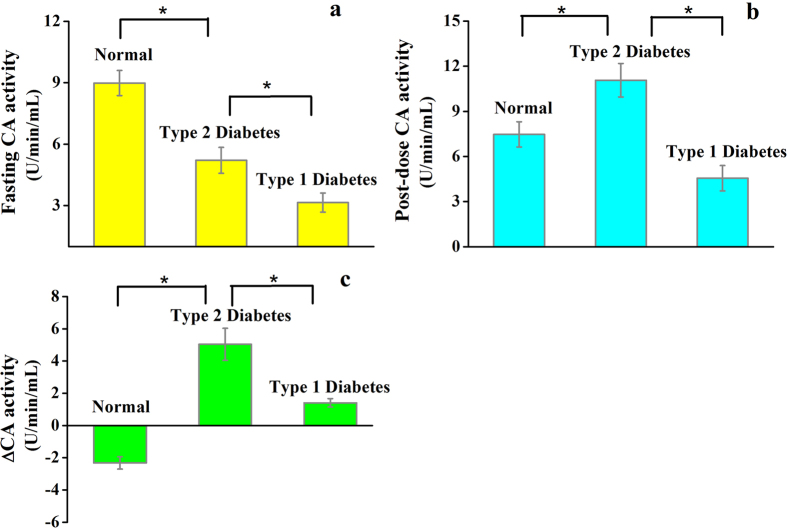
Pre-and post-dose erythrocytes CA activities during oral glucose tolerance test in non-diabetic control (NDC), type 2 diabetes (T2D) and type 1 diabetes (T1D). (**a**) Fasting CA activities [p < 0.01 for T1D (3.1 ± 1.4) versus T2D (5.2 ± 1.3) and NDC (8.9 ± 1.3)]. (**b**) Post-dose CA activities [p < 0.001 for T1D (4.5 ± 1.9) as compared with T2D (11.1 ± 2.8) and NDC (7.5 ± 1.36)]. *p < 0.01. Data are means ± SEM. (**c**) ∆CA activities for T1D (1.4 ± 0.26) as compared with T2D (5.03 ± 0.9) and NDC (−2.3 ± 0.4). *p < 0.01. Values are means ± SEM.

To investigate how carbonic anhydrase activity is associated with altered metabolism in T1D and T2D, we first estimated the total erythrocytes CA activity involving three primary isoenzymes of CA (i.e. CAI, CAII and CAIII), spectrophotometrically in the fasting state (Fig. 1a). The basal CA activity was found to be significantly lower in diabetic individuals as compared to non-diabetic controls (NDC, n = 27). However, in between T1D (n = 14) and T2D (n = 24), the individuals with T1D exhibited considerably lesser CA activity than T2D individuals.

Several earlier studies^11^,^12^ suggest that glycosylation is a negative regulator of erythrocytes CA activity in human body and the specific enzymatic activity of glycosylated CA (G-CA) was reported to be 40% of that of the unglycosylated CA. Therefore, glycosylation of CA may play an important role for the reduction of basal CA activities in T1D and T2D. In T1D, because of insulin deficiency, the erythrocytes are exposed to the highest levels of glucose-mediated environment, suggesting higher chance of glycosylation of CA to reduce its enzymatic activity more than T2D. These results suggest that the measurement of basal CA activity in erythrocytes may be an important parameter for the identification and classification of T1D and T2D.

To address the potential role of CA activity in response to glucose ingestion, we next determined the post-dose CA activities in both T1D and T2D. Our results (Fig. 1b) showed that post-dose CA activities were enhanced in diabetic subjects with respect to the basal CA activities during the 2 h-oral glucose tolerance test (2 h-OGTT). However, the enhancement of post-dose CA activity was significantly higher in T2D patients when compared to patients with T1D. Several lines of evidence^15^ suggest that the lack of erythrocyte receptor bound insulin creates an ionic imbalance within the erythrocytes cell membrane and subsequently alter the erythrocytes CA activity, indicating that in our observations the alterations of post-dose CA activities in individuals with T1D and T2D are likely to be the effect of ionic imbalance within the erythrocytes. However, our study demonstrates that the post-dose CA activity may also be a good candidate to distinguish T1D from T2D.

We next explored whether the absolute changes in erythrocytes CA activities between pre-and post-dose states (i.e. ∆CA) allow us to distinguish the exact state of the metabolic disorder. We observed marked differences in ∆CA activities in all individuals (Fig. 1c), suggesting a potential link between changes in erythrocytes CA activities and altered metabolism in diabetes mellitus. Our results also signify that changes in the activities of CA in human erythrocytes may contribute to the development of T1D and T2D. In view of these results, we also propose that measurements of ∆CA activities have an enormous potential to clearly distinguish between diabetic and non-diabetic subjects.

However, to investigate whether the alteration in CA activity in erythrocytes during the 2 h-OGTT could influence the rate of isotopic fractionation reaction during metabolism, we monitored the ^18^O/^16^O-stable isotope ratios of the major metabolite, CO_2_ in exhaled breath by using a laser-based high-resolution cavity-enhanced integrated cavity output spectroscopy (ICOS) technique. Here, we expressed the ^18^O/^16^O-isotope ratios of CO_2_ by the delta-over-baseline (DOB) with respect to the standard Vienna Pee Dee Belemnite, i.e. δ_DOB_^18^O‰ = [(δ^18^O‰)_t=t_ −(δ^18^O‰)_t=basal_].

**Figure 2:**
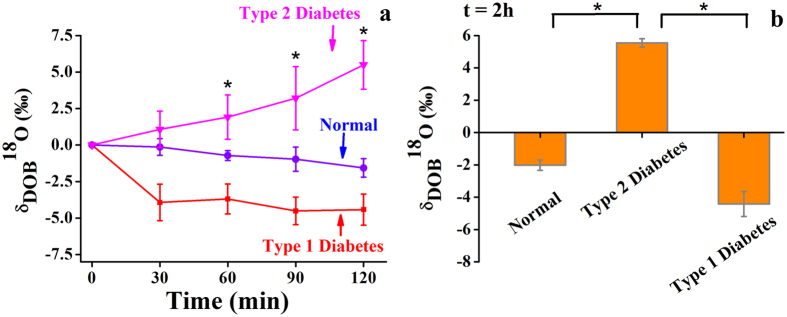
Excretion kinetics study of oxygen-18 isotopes in exhaled breath CO_2_ (expressed as δ_DOB_^18^O) for type 2 diabetes (T2D) and type 1 diabetes (T1D). (**a**) Breath δ_DOB_^18^O(‰) values are clearly distinguishable from 60 min to 120 min after glucose load in NDC, T2D and T1D. (**b**) δ_DOB_^18^O‰ for T1D (−4.4 ± 0.7) as compared with T2D (5.54 ± 0.2) and NDC (−2.9 ± 0.3). *p < 0.01. Values are means ± SEM.

Figure 2a shows the excretion kinetics patterns of ^18^O-isotopes of breath CO_2_ in NDC, T2D and T1D. In this investigation, T1D patients exhibited marked depletions of ^18^O-isotopes from the basal value as compared to NDC, whereas T2D patients manifested the highest isotopic enrichments of ^18^O-isotopes in their breath samples during the 2 h-OGTT, thus unveiling a potential link between the breath ^18^O-isotopic fractionations and altered metabolisms in T1D and T2D subjects, which has never been explored before. It is noteworthy that in non-insulin dependent T2D patients, the isotopic enrichments of ^18^O is possibly attributed to the effect of the enhancement of post-dose CA activity where it takes an important role to promote the isotopic exchange reaction during the metabolism to produce more ^18^O-isotopes in exhaled breath CO_2_.

However, in T1D subjects, although the post-dose CA activity was slightly higher than the basal value, but those subjects exhibited a significant depletion of ^18^O values in breath CO_2_ after glucose load. This is possibly due to the fact that in absence of insulin in T1D, the body switches to non-insulin dependent free fatty acids (FFAs) oxidation to supply energy for cellular work^16^. The FFAs are broken down in liver to produce ketone bodies which are then accumulated in bloodstream to lower its pH, thus making it acidic. As a result, the decrease of pH may facilitate the reverse hydrolysis of the isotopic fractionation reaction which may eventually reduce the production of ^18^O-isotope in breath CO_2_.

It is quite evident (Fig. 2b) that δ_DOB_^18^O‰ in exhaled breath was depleted in NDC and T1D subjects, whereas T2D exhibited significant enhancement of δ_DOB_^18^O‰ values after the 2 h-OGTT. Therefore, our evidences suggest that monitoring ^18^O/^16^O-isotopic exchange between ^12^C^16^O^16^O and H_2_^18^O influenced by the enzymatic activity of CA may be a new and robust route to selectively distinguish T1D from T2D and thus breath ^12^C^18^O^16^O might be considered as a potential biomarker for the precise evaluation of T1D and T2D in a non-invasive way.

**Figure 3:**
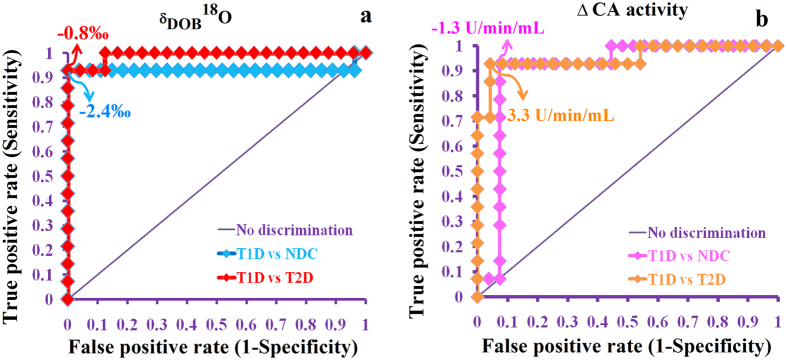
Clinical feasibility of δ_DOB_^18^O (‰) and ∆CA activity (U/min/mL) measurements. The optical diagnostic cut-off points were estimated by utilizing the receiver operating characteristic (ROC) curves analysis. (**a**) δ_DOB_^18^O‰ < −2.4‰ for T1D; δ_DOB_^18^O ‰ > −0.8 for T2D, whereas (**b**) The −1.3 < ∆CA activity < 3.3 U/min/mL for distinguishing T1D from T2D. Data are means ± SD.

However, to establish the widespread clinical applicability of ^18^O-isotopes of breath CO_2_, we attempted to estimate the optimal diagnostic cut-off values of δ_DOB_^18^O‰ by utilizing the receiver operating characteristic (ROC) curve analysis (Fig. 3). From the ROC curve analysis, the individuals with δ_DOB_^18^O‰ < −2.4‰ were considered as T1D, whereas subjects with δ_DOB_^18^O‰ > −0.8‰ were suggested to be T2D and these corresponded to the diagnostic sensitivity and specificity of ~92% and ~100%, respectively. Similarly, a diagnostic cut-off point of −1.31 < ΔCA < 3.3 U/min/mL was considered as T1D to specifically distinguish T1D from T2D corresponding to the similar levels of diagnostic sensitivity and specificity. Taken together, our findings therefore suggest a potential unifying strategy for precise classification and diagnosis of T1D and T2D because carbonic anhydrase (CA) is a ubiquitous enzyme in nature present in the human body and occurs in most cells and tissues.

**Figure 4:**
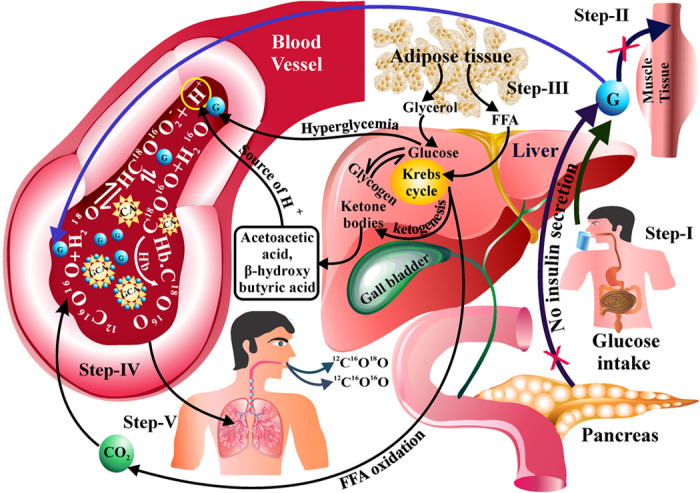
Proposed physiological pathways of oxygen-18 isotopic fractionation in diabetes. At fasting state, carbonic anhydrase (CA) loses its enzymatic activity in diabetes subjects due to formation of excess glycosylated carbonic anhydrase molecules (G-CAs) in erythrocytes. The decrease in enzymatic activity of CA is the maximum in T1D as erythrocytes are exposed to the highest level of glucose-mediated environment in T1D than T2D. When a dose containing of 75 g of normal glucose is administered (step-I), due to lack of insulin, the exogenous glucose cannot enter into muscle tissue (step-II) in T1D. Therefore, the non-insulin dependent free fatty acids (FFAs) oxidation is facilitated to produce ketone bodies during metabolism in liver (step-III).This may alter the reverse isotopic fractionation reaction to deplete the ^18^O-isotope in exhaled breath CO_2_ at post-dose state (step-IV to step-V).

However, in order to understand the mechanisms underlying the alteration of breath ^18^O-isotopic fractionations regulated by the CA activity, we finally elucidated the possible metabolic pathways to explicate our findings (Fig. 4). It is known that insulin promotes the cellular glucose uptake and removes the excess glucose from the blood. In T1D individuals, due to lack of insulin, glucose molecules build up in the bloodstream to create hyperglycemia, suggesting the possibility of erythrocytes CA to be exposed in higher level of glucose-mediated environment, which in turn makes a higher glycosylated CA (G-CA) enzymes than that in T2D individuals. Therefore, the maximum suppression of basal CA activity in T1D is possibly attributed to the formation of large number of G-CAs in erythrocytes at fasting state. After overnight fasting, when a test meal containing 75 g of normal glucose is administered, the majority of glucose disposal takes place in insulin-dependent peripheral tissues (primarily muscle tissue), whereas adipose tissue accounts for only small amount of exogenous glucose uptake. In case of T1D, the lack of insulin may be responsible to facilitate the non-insulin dependent free fatty acids (FFAs) oxidations to supply the metabolic fuel. This may accumulate the excess ketone bodies in the blood. This may be responsible to alter the rate of the reverse isotopic fractionation reaction to deplete the ^18^O-isotope in exhaled breath CO_2_ at the post-dose state in T1D.

Here, we have elucidated a possible mechanism underlying the cause of change in erythrocytes CA activity during the metabolism in T1D and T2D. However, many important gaps remain in our understanding of the physiological pathways regarding the oxygen-18 isotopic exchange and metabolic pathways involved during metabolism. But, our method shows a potential perspective of exhaled breath analysis as the innovative diagnostic tool in future days. Although organ specific metabolic study is not available from these experiments, but future studies that include the experimental animal models are needed to be ensured how different organs are associated with the oxygen-18 isotopic fractionations during the metabolism in T1D.

## Conclusion

Our findings reveal a fundamental mechanism underlying the potential role of erythrocytes CA activity in the pathogenesis of T1D and T2D. We have also taken a major step towards unravelling a missing link between CA activity in human erythrocytes and ^18^O- isotopic fractionations in human breath CO_2_ which has never been explored before. Our studies suggest that breath ^12^C^18^O^16^O and erythrocytes CA activity could be used as potential biomarkers for precise classification and diagnosis of T1D from T2D in a more robust and better way compared to the traditional methods which usually utilize one or more antibody testing. Another salient advantage of our methodology is that it may assist for non-invasive early evaluation of those individuals who are now T2D with higher rate of genetically driven beta cell apoptosis, but are highly susceptible to exhibit metabolic transition from T2D to T1D in future days. Moreover, new insights into the linkages between ^18^O of breath CO_2_ and CA activity in erythrocytes are fostering to devise new and better approaches to understanding the pathophysiology of T1D and T2D along with new pharmacological targets for the treatment and prevention of the world’s most common long-lasting metabolic diseases.

## Methods

### Subjects

Sixty five subjects (n = 27 non-diabetic control, n = 24 type 2 diabetes and n = 14 type 1 diabetes) were enrolled for the study. The screening procedure was comprised of the measurements of fasting and PP blood glucose concentrations, HbA1c (%), fasting and PP insulin levels etc. All participants went through a medical history check by the physicians prior to recruitment. The exclusion criteria included the presence of interstitial lung disease, hypertension, chronic respiratory disorder, smoking or abuse of drugs and alcohol, taking any medication which could alter the carbohydrate metabolism etc. Based on the HbA1c (%), 2 h-OGTT and autoantibody testing, the study subjects were allocated into three different groups: non-diabetic control (NDC) (2 h-OGTT < 140 mg/dl and HbA1c < 5.7), newly diagnosed type 2 diabetes (T2D) (2 h-OGTT > 200 mg/dl and HbA1c ≥ 6.5) and type 1 diabetes (T1D) (HbA1c ≥ 6.5 and GAD-65 antibody ≥ 10 IU/mL). The clinical characteristics parameters are described in Table 1. The whole protocol was approved by the Institutional Ethics Committee of Vivekananda Institute of Medical Sciences (Registration No. ECR/62/Inst/WB/2013). The methods were carried out in accordance with the approved guidelines of the ethics committee. Informed written consent was taken from each subject before participating in the study.

### Study protocol

After overnight fasting, breath and blood samples were taken from each participant. The subjects were then instructed to ingest a test meal containing 75 gm normal glucose dissolved in 150 mL water. Post-dose end-tidal breath samples were collected in breath samples collection bags (QUINTRON, USA, SL No. QT00892) at 30, 60, 90 and 120 minutes and analyzed immediately by laser-based ICOS spectrometer to measure the isotopes of carbon dioxides in exhaled breath. After 2 h of glucose load, the blood samples were collected again to determine the erythrocytes carbonic anhydrase activities and post-dose blood parameters.

### Biochemical analysis

The fasting and post-dose plasma blood glucose concentrations were estimated spectrophotometrically (2300 STAT Plus Glucose Analyzer). The insulin levels were estimated by using monoclonal antibody coated immunoassay DIAsource INS-EASIA Kits (DIAsource ImmunoAssays S. A. Rue du Bosquet, 2, B-1348 Louvain-la-Neuve, Belgium). The glycosylated hemoglobin (HbA1c %) was measured by HPLC method. The carbonic anhydrase activity was estimated by utilizing UV-Vis spectrophotometer (Shimadzu UV-2600 Spectrophotometer).

### Blood sample preparation

The collected venous blood samples were centrifuged at 4,000 rpm for 5 minutes to remove the plasma and the buffy coats. Then the erythrocytes were washed with 0.9% NaCl solution. The whole sample mixture was allowed to spin at 4,000 rpm for 20 minutes. Thereafter, the erythrocytes were lysed with ice cold distilled water to prepare hemolysate. Next, the hemolysate was again centrifuged at 10,000 rpm for 10 minutes to separate the ghost cells. The supernatant liquid was collected and used for carbonic anhydrase activity measurement.

### Total erythrocyte carbonic anhydrase activity measurement

The total esterase activity of carbonic anhydrase was estimated by the method described by Armstrong *et al.*^21^ with the modifications described by Parui *et al.*^15^. In this method, the carbonic anhydrase activity was measured spectrophotometrically from the hydrolysis rate of p-nitrophenyl acetate to p-nitrophenol. Many proteins, present in hemolysate have the ability to hydrolyse the p-nitrophenyl acetate to produce the p-nitrophenol. Therefore, a specific inhibitor of carbonic anhydrase i.e. acetazolamide (AZM), was used to inhibit the carbonic anhydrase enzymes selectively in erythrocytes. From the difference of optical densities in presence and absence of AZM, the carbonic anhydrase activity was calculated. The assay system was comprised of 100 μL hemolysate placed in a 1 cm cuvette in presence of TRIS buffer (pH = 7.4). The change of absorbance was measured by a UV-Vis spectrophotometer over a time period of 3 min at 348 nm before and after addition of hemolysate. The net activity was calculated from the following formula:


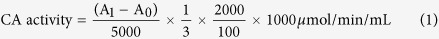


where A_1_ and A_0_ are the absorbance at 3 minutes and 0 minute after addition of hemolysate respectively (equation 1). The molar absorptivity of p-nitrophenol is 5000 M^−1^ cm^−1^. The carbonic anhydrase activity was calculated from the amount of p-nitrophenol released per minute per mL of hemolysate. The activity was normalised to 4.5 × 10^9^ cells/mL.

### Integrated Cavity Output Spectroscopy for breath analysis

Cavity enhanced absorption technique is a powerful technique for measurement of gases and their isotopes in ultra-low concentration with high-precision^17^,^18^. In our study, we utilized a carbon dioxide isotope analyzer (CCIA 36-EP, LGR, USA) exploiting cavity enhanced spectroscopy based on off-axis cavity output spectroscopy (ICOS) to measure the ^18^O/^16^O isotope ratios in exhaled breath CO_2_. The technical details of the ICOS method have been described elsewhere^19^,^20^. The ICOS system is comprised of a near infrared continuous wave distributed feedback diode laser operating at ~2.05 μm. The concentrations of ^12^C^18^O^16^O and ^12^C^16^O^16^O isotopes in breath samples were calculated using Beer’s law. The absorption spectra of ^12^C^18^O^16^O, ^12^C^16^O^16^O and ^13^C^16^O^16^O isotopes were recorded at the wave numbers of 4874.178 cm^−1^, 4874.448 cm^−1^ and 4874.086 cm^−1^ respectively by tuning the laser frequency to scan over 20 GHz across the P(36), R(28) and P(16) ro-vibrational lines in the (2,0^**0**^,1) ← (0,0^**0**^,0) vibrational combination band of the CO_2_ molecule. The enrichment of ^12^C^18^O^16^O isotope in exhaled breath is represented by δ^18^O‰ = (R_sample_/R_standard_ −1) × 1000, where R_sample_ is the ^18^O/^16^O isotope ratio of breath CO_2_ and R_standard_ is the international standard Pee Dee Belemnite (PDB) value (0.0020672). We have estimated the accuracy and precision of δ_DOB_^18^O‰ measurements by using the standard NOAA air tank (Supplementary Table 1). The precision was determined to be ±0.11‰ for the δ_DOB_^18^O‰ measurements in our study (Supplementary Table 2).

### Statistical method

In the current study, Origin Pro 8.0 (Origin Lab Corporation, USA) and Analyse-it Method Evaluation software (Analyse-it Software Ltd, UK, version 2.30) were utilized for the statistical analyses. Normality test was performed to check whether the data were normally distributed or not. Based on the normality test results, one way analysis of variance (ANOVA) for normally distributed data and Kruskal-Wallis test and Mann-Whitney test for non-normal distributed data were performed to compare the data sets. Data were considered to be statistically significant when the two-sided p-value was less than 0.05. To check the diagnostic efficacy to differentiate the diseased and non-diseased states, receiver operating characteristic curves (ROC) were drawn for estimating the optimal cut-off values. The cut-off values of δ_DOB_^18^O‰ and ∆CA activities were corresponded to the data points which showed the maximum sensitivity and specificity for the diagnosis of the disease (supplementary Table 3). The p value was also calculated for demographic study (supplementary Figure 1). The positive and negative predictive values were also calculated for the study.

## Additional Information

**How to cite this article**: Ghosh, C. *et al.* Targeting erythrocyte carbonic anhydrase and ^18^O-isotope of breath CO_2_ for sorting out type 1 and type 2 diabetes. *Sci. Rep.* **6**, 35836; doi: 10.1038/srep35836 (2016).

## Supplementary Material

Supplementary Information

## Figures and Tables

**Table 1:** Clinical parameters of the study subjects. Data are expressed as mean ± SD.

Parameters	Non-diabetic control (NDC) (n = 27)	Type 2 Diabetes (T2D) (n = 24)	Type 1 Diabetes (T1D) (n = 14)	p values
Sex (M/F)	16/11	15/9	8/6	
Age (Years)	31.7 ± 8.9	36.2 ± 11.7	21.7 ± 13.8	<0.001*
Weight (kg)	61.2 ± 7.2	60.1 ± 5.8	47.1 ± 11.8	<0.001*
BMI (kg/m^2^)	23.9 ± 2.4	24.6 ± 2.3	19.4 ± 2.9	<0.001*
Fasting Plasma Glucose (mg/dL)	87.7 ± 9.8	132.08 ± 12.2	272.6 ± 107.6	<0.001*
Fasting Plasma Insulin (μIU/mL)	9.68 ± 3.1	5.2 ± 1.3	1.1 ± 0.7	<0.001*
2-hr Post-dose Plasma Glucose (mg/dL)	126.6 ± 10.3	269.8 ± 37.9	452 ± 99.9	<0.001*
HbA1c (%)	5.2 ± 0.2	7.5 ± 0.5	11.3 ± 2.9	<0.001*
Fasting CA Activity (U/min/mL)	8.9 ± 1.3	5.2 ± 1.3	3.1 ± 1.4	<0.001*
Post-dose CA Activity (U/min/mL)	7.5 ± 1.36	11.1 ± 2.8	4.5 ± 1.9	<0.001*

*Represents statistically significant differences (p < 0.05) among non-diabetic control (NDC), type 2 diabetes (T2D) and type 1 diabetes (T1D). The abbreviations M and F stand for male and female, respectively.
