# Supplementary material for: Targeting erythrocyte carbonic anhydrase and 18O-isotope of breath CO2 for sorting out type 1 and type 2 diabetes
Source: Sci Rep. 2016 Oct 21;6:35836. doi: 10.1038/srep35836 (PMC5073312; doi:10.1038/srep35836)

Supplementary information for

Targeting erythrocyte carbonic anhydrase and ^18^O-isotope of breath CO_2_ for sorting out type 1 and type 2 diabetes

Chiranjit Ghosh, Santanu Mandal, Gourab D Banik, Abhijit Maity, Prabuddha Mukhopadhyay, Shibendu Ghosh, and Manik Pradhan*

*To whom correspondence should be addressed.

Dr. Manik Pradhan, *Email:* [manik.pradhan@bose.res.in](mailto:manik.pradhan@bose.res.in)

**Supplementary Table 1:**

Test Accuracy. Results of δ^18^O (‰) measurements of nine flasks filled from a certified standard NOAA air tank (Serial No.CB10073).

| **Flask** | **δ^18^O (‰) measured by**  **ICOS method** |
| --- | --- |
| Flask-a | -1.11 |
| Flask-b | -0.88 |
| Flask-c | -0.76 |
| Flask-d | -1.19 |
| Flask-e | -1.28 |
| Flask-f | -1.08 |
| Flask-g  Flask-h  Flask-i | -1.32  -1.12  -0.93 |
| Avg. | -1.07 |
| NOAA | -1.00 |
| **Diff.** | 0.07 |

**Supplementary Table 2:**

Determination of test precision of δ_DOB_^18^O‰ measurements by ICOS method. Pre-and post-dose breath samples were taken from a subject and analyzed by the ICOS spectrometer. Test precision was estimated from the eight consecutive measurements of each set (pre-and post-dose breath samples). The precision was calculated from the standard error in the measurements of δ_DOB_^18^O values.

| **(δ^18^O)_measurement_** | **Difference (δ_DOB_^18^O ‰) measured**  **by ICOS method** |
| --- | --- |
|    (δ^18^O)test_1-(δ^18^O)test_2 ‰ | 5.09 |
|   (δ^18^O)test_3-(δ^18^O)test_4 ‰ | 4.66 |
|    (δ^18^O)test_5-(δ^18^O)test_6 ‰ | 4.61 |
|  (δ^18^O)test_7-(δ^18^O)test_8 ‰ | 5.23 |
|   (δ^18^O)test_9-(δ^18^O)test_10 ‰ | 4.68 |
|    (δ^18^O)test_11-(δ^18^O)test_12 ‰ | 4.43 |
|    (δ^18^O)test_13-(δ^18^O)test_14 ‰     (δ^18^O)test_15-(δ^18^O)test_16 ‰ | 5.12  4.89  Precision= ± 0.1 |

**Supplementary Table 3:**

Estimation of diagnostic parameters related to cut-off values of ∆CA activity (U/min/mL) and δ_DOB_^18^O (‰) values by ICOS method for screening of type 1 diabetes (T1D) and type 2 diabetes (T2D). AUC: area under the curve; PPV: positive predictive value; NPV: negative predictive value*.*

| **Groups** | **Cut-off points of ΔCA activity**  **(U/min/mL)** | **Sensitivity** | **Specificity** | **PPV** | **NPV** | **AUC** | **Accuracy** |
| --- | --- | --- | --- | --- | --- | --- | --- |
| NDC  vs  T1D | -1.31 | 92% | 92% | 86.0% | 97% | 0.905 | 92.1% |
| T1D  vs  T2D | 3.3 | 92.9% | 95.8% | 92.0% | 96.0% | 0.952 | 94.7% |

| **Groups** | **Cut-off points of δ^18^O (‰)** | **Sensitivity** | **Specificity** | **PPV** | **NPV** | **AUC** | **Accuracy** |
| --- | --- | --- | --- | --- | --- | --- | --- |
| T1D  vs  T2D | -0.8 | 92.9% | 100.9% | 100.0% | 96% | 0.931 | 97.3% |
| T1D  vs  NDC | -2.4 | 92.9% | 100% | 100.0% | 96.0% | 0.991 | 96.4% |

**Supplementary Figure 1:**

Determination of clinical validity of the test within the different populations represented as category 1, category 2 and category 3.


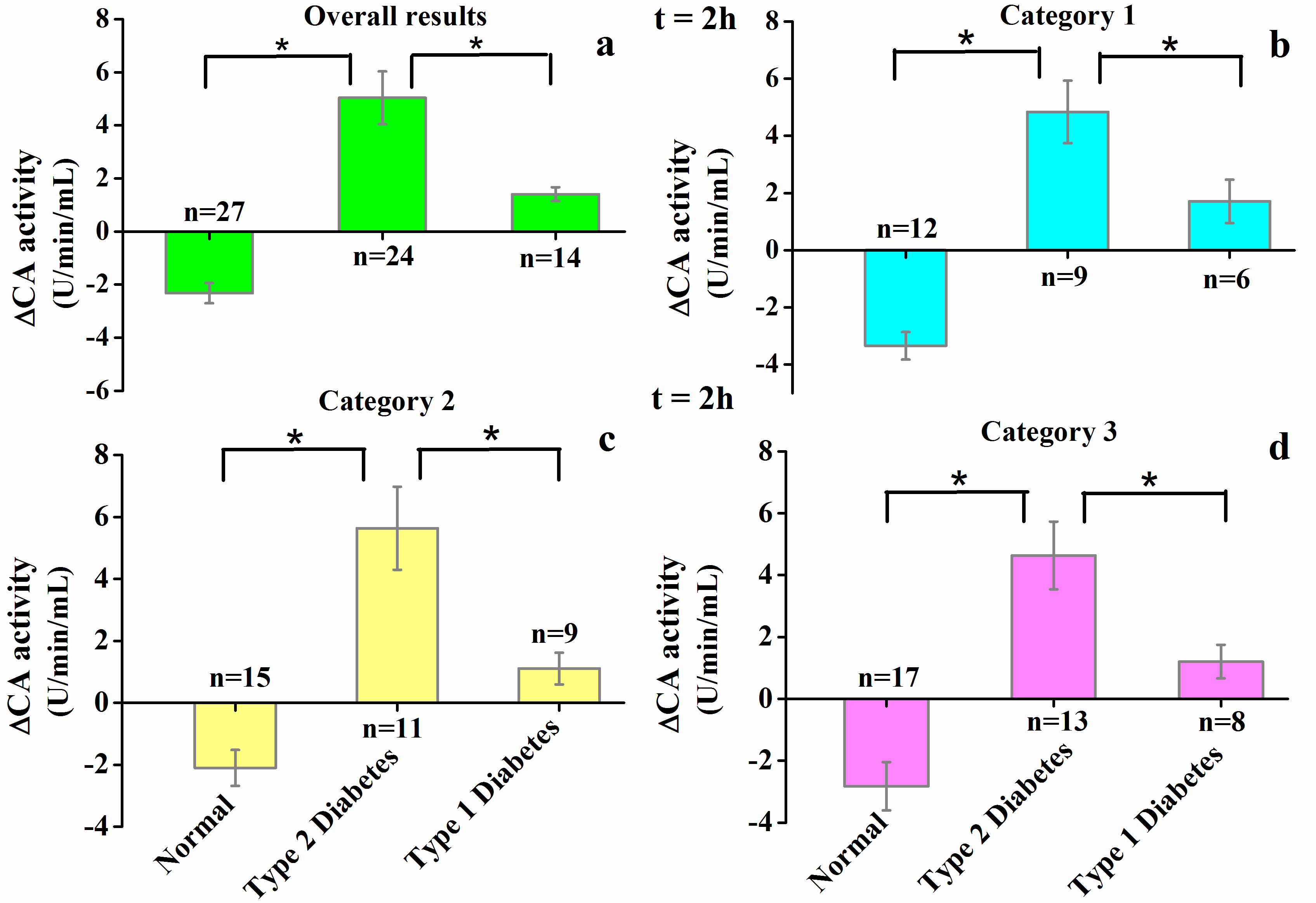


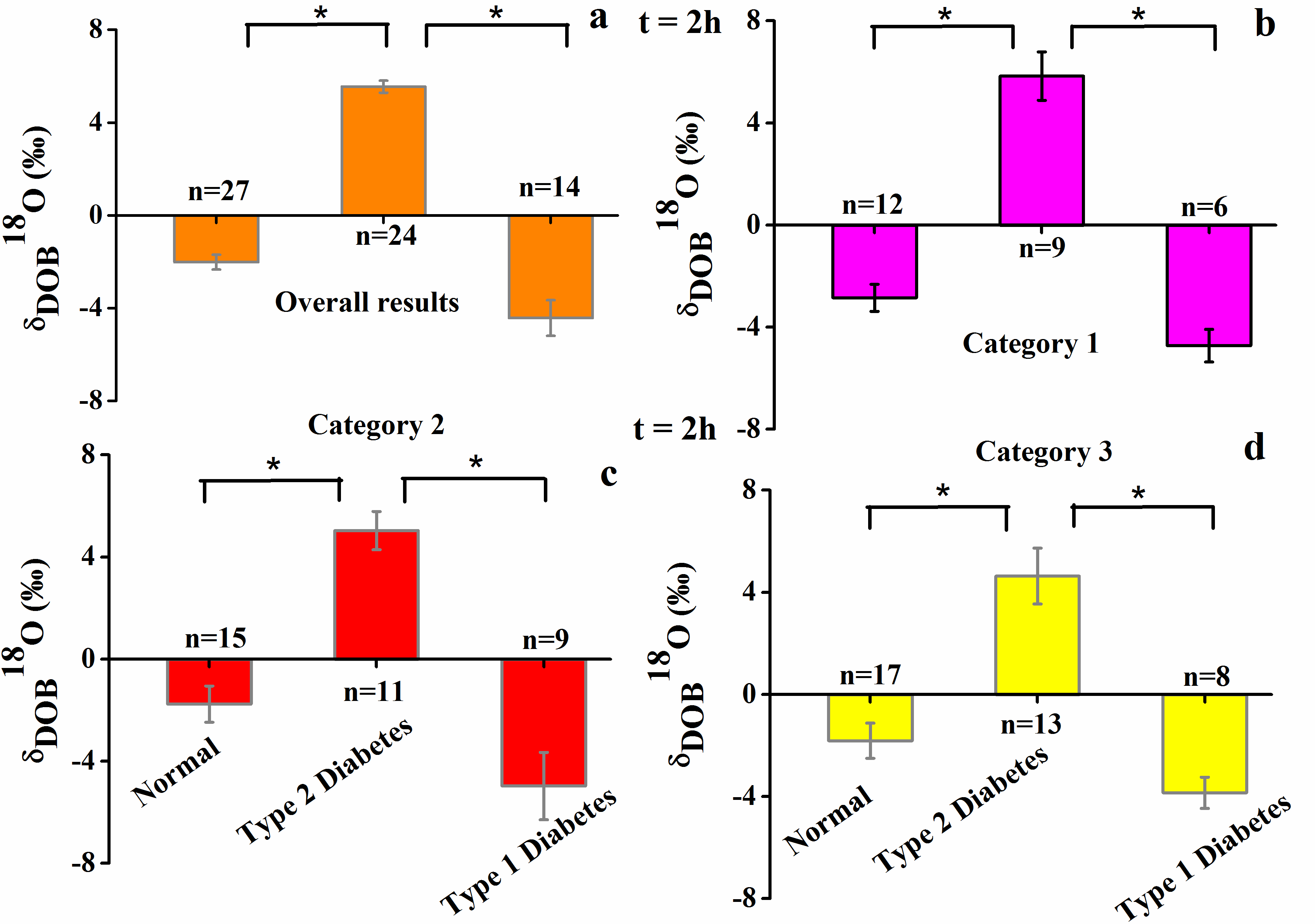

Supplement: Supplementary Information [file srep35836-s1.doc]
